# Spinal afferent innervation in flat-mounts of the rat stomach: anterograde tracing

**DOI:** 10.1038/s41598-023-43120-y

**Published:** 2023-10-18

**Authors:** Jichao Ma, Duyen Nguyen, Jazune Madas, Andrew M. Kwiat, Zulema Toledo, Ariege Bizanti, Nicole Kogut, Anas Mistareehi, Kohlton Bendowski, Yuanyuan Zhang, Jin Chen, De-Pei Li, Terry L. Powley, John B. Furness, Zixi Cheng

**Affiliations:** 1 Burnett School of Biomedical Sciences, College of Medicine, University of Central Florida, Orlando, FL 32816 USA; 2 Department of Medicine, Center for Precision Medicine, School of Medicine, University of Missouri, Columbia, MO 65212 USA; 3 Department of Psychological Sciences, Purdue University, West Lafayette, IN 479062 USA; 4 Department of Anatomy and Physiology, University of Melbourne, and Florey Institute of Neuroscience and Mental Health, Parkville, VIC Australia

**Keywords:** Anatomy, Gastrointestinal system, Nervous system

## Abstract

The dorsal root ganglia (DRG) project spinal afferent axons to the stomach. However, the distribution and morphology of spinal afferent axons in the stomach have not been well characterized. In this study, we used a combination of state-of-the-art techniques, including anterograde tracer injection into the left DRG T7-T11, avidin–biotin and Cuprolinic Blue labeling, Zeiss M2 Imager, and Neurolucida to characterize spinal afferent axons in flat-mounts of the whole rat stomach muscular wall. We found that spinal afferent axons innervated all regions with a variety of distinct terminal structures innervating different gastric targets: (1) The ganglionic type: some axons formed varicose contacts with individual neurons within myenteric ganglia. (2) The muscle type: most axons ran in parallel with the longitudinal and circular muscles and expressed spherical varicosities. Complex terminal structures were observed within the circular muscle layer. (3) The ganglia-muscle mixed type: some individual varicose axons innervated both myenteric neurons and the circular muscle, exhibiting polymorphic terminal structures. (4) The vascular type: individual varicose axons ran along the blood vessels and occasionally traversed the vessel wall. This work provides a foundation for future topographical anatomical and functional mapping of spinal afferent axon innervation of the stomach under normal and pathophysiological conditions.

## Introduction

In the past decades, neuroscience has made tremendous progress in clarifying and interpreting how our five sensory systems (somatosensation, vision, audition, olfaction, and taste) interact with the external world (exteroception). In contrast, limited information is available regarding the nervous systems that detect, represent, and interpret sensory information from the internal organs (interoception)^1–4^. Based on the available findings, the NIH Blueprint for Neuroscience Research recently identified a wide range of topics crucial for the future of interoception research, including the very limited research in the visceral afferent innervation of the internal organs. For example, abdominal pain is associated with gastrointestinal (GI) tract peripheral nociception but the anatomical characterization of neural circuitry in the GI tract that underlies peripheral nociceptive processes is not well characterized. GI nociceptive processes are primarily mediated by visceral afferent neurons in the spinal dorsal root ganglia (DRG) and, to a lesser extent, by the vagal nodose ganglion (VNG)^5–7^. Previous researchers assessed the vagal afferent innervation of the stomach and found that vagal afferent axons form two distinct terminal structures: intraganglionic laminar endings (IGLEs; tension receptors) in the myenteric plexus and intramuscular arrays (IMAs; stretch receptors) in the longitudinal and circular muscles^8–13^. Studies in the past have also focused on the vagal and sympathetic efferent innervation of the proximal digestive tract^8,14–18^. In contrast to vagal afferents/efferents whose nerve endings have been well described in the GI tract using anterograde tracing^12,18–20^, a comprehensive characterization of spinal afferent innervation of the stomach is limited^21^. This limitation results from a dearth of advanced techniques that allow us to specifically label, visualize, trace, and digitize individual spinal afferent terminals in the GI tract. Within the GI tract, spinal/vagal afferent, sympathetic/vagal efferent and the intrinsic enteric nervous system coexist and form a complex circuitry. This circuitry of extrinsic innervation to the gut cannot be completed without the involvement of spinal afferents, hence this is a missing puzzle piece to build a more comprehensive understanding of visceral sensory/motor pathways^22–25^.

Spinal afferents detect and convey sensory information to the central nervous system for processing^26^. At the effector tissues, the nerves make contacts by forming varicosities that contain synaptic vesicles^27^. These vesicles release neurotransmitters upon interaction with receptors that are distributed widely on the targets’ surface, exerting their functions on the targets. The innervation of spinal afferent axons in different targets observed in this study, therefore, is the neuroanatomical evidence that such innervation may imply functional significance on the stomach.

Previously, the distribution and morphology of peptidergic axons in the mouse stomach was determined using immunohistochemical labeling of substance P (SP) and calcitonin gene-related peptide (CGRP)^28–30^. SP and CGRP are conventionally used as markers for nociceptive nerves. Although many SP-IR and CGRP-IR axons in the stomach are from the DRG, other sources such as the VNG and myenteric neurons also contribute to these axon populations^28–32^, hence the distribution and the morphological features of SP-IR and CGRP-IR axons cannot exclusively represent the DRG afferent projections to the stomach. Noticeably, previous studies utilized tracer injections into the thoracic DRG of cats and identified spinal afferent axons at the esophagogastric junction that ramified around myenteric neurons, innervated the circular muscle fibers, and traveled along the blood vessels^27,33,34^. However, the whole structure of these labeled fibers could not be completely determined due to the use of sectioned tissues that severed the continuity and integrity of nerve fibers. To overcome this major drawback, we injected anterograde tracer dextran biotin (DB, biotinylated dextran amine) into the left DRG (T7–T11) and processed the whole stomach muscular layers as flat-mounts to specifically label the spinal afferent axons and determine their terminal structures in different regions. DB has been previously proven to be reliable in labeling both visceral afferents^18,35,36^ as well as axons and terminals throughout the central nervous system^37–39^, hence the use of this tracer in our study.

## Materials and methods

### Animals and ethical statement

All procedures were approved by the University of Central Florida Animal Care and Use Committee (HURON PROTO202000115) and strictly followed the guidelines established by the National Institutes of Health (NIH) and ARRIVE 2.0. Four to five-month-old male Sprague–Dawley rats (n = 20; RRID: RGD_737903; Envigo, Indianapolis, IN) were housed in an animal room at which the dark/light cycle was set to 12/12 h and water and food were supplied ad libitum.

### Neural tracer injections

After a 2-week acclimation period in the colony, overnight-fasted rats were anesthetized with isoflurane (#029405, Covetrus North America, Dublin, OH) inhalation (4% in oxygen for induction, 2–3% for maintenance). Hind paw pinch withdrawal reflexes were checked as indicators of the depth of anesthesia. The procedures were described previously^23^. Briefly, whilst under anesthesia, the rat was placed in a ventral decubitus position, and a 5–6 cm long incision was made along the midline of the dorsal surface. Left paraspinal muscles were separated from the transverse process by blunt dissection to expose the lateral aspect of the thoracic vertebrae. Five DRG, from T7 to T11, were then exposed by drilling holes dorsally at T7-T11 articular processes covering the intervertebral foramina.

For the DRG injection, a 10-μl syringe (Cat. #80300; Hamilton Company, Reno, NV) was used to aspirate 2-μl of lysine-fixable Dextran Biotin (DB) solution consisting of a 1:1 mixture of 3 K and 10 K MW dextrans in ultrapure water (final concentration 15% dextran biotin consisting of 7.5% D7135 (3 K) and 7.5% D1956 (10 K); Invitrogen, Carlsbad, CA). This 2-μl solution was then transferred to a glass micropipette (outer diameter: 1.5 mm, inner diameter 1.12 mm; cat. #TW150-4; World Precision Instruments, Sarasota, FL) that was pulled on a Flaming Brown micropipette puller (model P-87; Sutter Instruments, Novato, CA) with tip diameter of approximately 5 μm. The micropipette was inserted at an approximate 45-degree angle relative to the spine into the posterior side of DRG, and the tracer was slowly injected as the needle gradually retracted in a stepwise fashion within the ganglion. The micropipette was kept in place for 1 min during each injection before withdrawal to ensure the infusion pressure within the DRG had dissipated. All five DRG (T7-T11) on the left side were injected in each rat. After the injection, the paraspinal muscles and skin were closed using interrupted sutures. Rats were placed on a heating pad at 37 °C for recovery and regaining of their righting reflexes prior to being returned to their home cages. To minimize postsurgical discomfort and pain, buprenorphine (0.01 mg/kg body weight; s.c., Par Pharmaceuticals, Chestnut Ridge, NY) was administered during operation and then again once every 24 h over the following 72 h postsurgery. Also, to prevent infection, a single dose of penicillin (50,000 IU/kg body weight; i.m., NorBrook, Newry, UK) was given. Both body weight and intake of food and water were monitored throughout the duration of the recovery period to ensure that rats did not experience any postsurgical malaise.

### Tissue fixation and stomach dissection

Sixteen days following survival surgery, the rats were weighed and deeply anesthetized with isoflurane (5%). Upon the absence of response to hind-paw pinching, 0.3 mL of heparin was injected into the left ventricle to prevent blood coagulation followed by a cut to the inferior vena cava. The animal was perfused through the left ventricle of the heart with 500 mL of physiological saline at 37 °C and then with 500 mL of 4% paraformaldehyde in phosphate-buffered saline (0.1 M PBS, pH 7.4) at 4 °C. In order to shape the stomach in normal distension at the time of fixation, a catheter was slowly intubated into the stomach and 10 mL of physiological saline at 37 °C was infused into the stomach. After the perfusion, the whole stomach was removed from the abdominal cavity by introducing cuts to the distal esophagus and the proximal duodenum. Next, a cut along the lesser and greater curvature was made to yield two equal stomach halves. The chyme was rinsed off to expose the mucosa. Eight hours into post-fixation with paraformaldehyde, the whole mount muscle wall of the stomach was separated from the gastric mucosa and submucosa using forceps. The muscle wall includes the longitudinal muscle, myenteric plexus, and circular muscle. The tissue processing protocol has been thoroughly described in^28^.

### DAB and cuprolinic blue staining

Similar to the protocol that was previously used for the rat stomach^17^, all steps of tracer processing and neuronal counterstaining of whole mount tissue were done in room temperature (~ 22 °C), on a shaker, and with the tissue free floating. The samples were washed 6 times × 5 min each in phosphate-buffered saline (PBS; 0.1 M, pH = 7.4), followed by a 30-min soaking in methanol:hydrogen peroxide block (4:1 ratio) to inactivate endogenous peroxidase. Following additional PBS washes, the tissues were soaked in a PBS solution containing 0.5% Triton X-100 and 0.08% sodium azide NaN_3_ for 5 days to facilitate reagent penetration. The samples were rinsed in PBS 6 times × 5 min each, followed by a one-hour incubation in avidin/biotin peroxidase complex (PK-6100; Vectastain Elite ABC Kit, Standard; Vector Laboratories Inc., Burlingame, CA). The DB-filled spinal afferent axons were visualized using diaminobenzidine (DAB), a traditional dye that produces a golden-brown color on labeled axons (Cat# D5905, Sigma-Aldrich, Inc., St. Louis, MO). Samples were then rinsed in PBS 6 times × 5 min each, soaked in DAB solution for 5 min, and rinsed in distilled water 6 times × 5 min. The pan-neuronal marker Cuprolinic Blue (CAS# 41276-95-3, American Elements, Los Angeles, CA) which binds to RNA in the cytoplasm of neurons^40^ was used to counterstain the enteric neurons in another group of samples (n = 6). Briefly, the samples were rinsed in distilled water and then incubated in 0.5% Cuprolinic Blue solution which dissolved in 0.05 M sodium acetate buffer containing 1.0 M MgCl_2_ (pH 4.9) for 2 h in a humidified slide warmer (38 °C). Next, the samples were rinsed in distilled water and incubated in 0.05 M sodium acetate buffer containing 1.0 M MgCl_2_ (pH 4.9) for 2 min, followed by a repeating rinse in distilled water. After the staining process, samples were mounted on slides, flattened under lead blocks for 6 h, air-dried overnight in a fume hood, dehydrated in an ascending concentration of alcohol (75%, 95%, 100%, and 100%), and cleared in xylene. Coverslips were used to cover the tissue after applying DPX mounting medium (317616; D.P.X.; Millipore Sigma, Burlington, MA).

### Spinal afferent axon screening and tracing

A Nikon eclipse 80i upright microscope (Lens: 20X, NA 0.5) with brightfield optics was first used to systematically examine all tissue samples. Once a DB-labeled spinal axon was identified, three criteria were used to inspect the quality of the axon: (a) adequate labeling, (b) completeness of the axon, and (c) minimal tissue artifacts such as folds and tears. All spinal axons were categorized based on their relative locations (e.g., fundus, corpus, antrum) and gastric targets (e.g., myenteric ganglia, longitudinal muscle sheet, circular muscle sheet, blood vessels). The spinal axons that satisfied all criteria were re-evaluated using a higher magnification lens (40X, NA 0.75) to assess the morphology of any intertwined neighboring individuals that could potentially lead to the failure of distinction and digitization of the entire tracing of a single spinal afferent axon. Such neighboring spinal axons were removed from the inventory, and the axons that satisfied all qualifications were marked and ready to be digitized.

Axon tracing, digitization, and analysis were performed using Neurolucida software (RRID:SCR_001775; MicroBrightField, Williston, VT, http://www.mbfbioscience.com/neurolucida). The software controlled the motorized stage of a Zeiss Axio Imager M2 microscope (RRID:SCR_018876, Oberkochen, Germany) equipped with brightfield optics and a long-working-distance 40X objective lens (NA 0.75). All spinal afferent axons were traced in real time and in their original 3-D space. While tracing, a set of criteria was used to identify the parent axon: A fiber segment that is long, large in diameter, and a first bifurcation point of less than 180 degrees (mostly less than 90 degrees). The “daughter” branches followed the same pattern of bifurcations. The tracing file was then saved as .xml.

The Neurolucida system was also used to locate and outline the myenteric ganglia on which the spinal afferent axons formed terminals or where the axon processes coursed in close proximity with and on the same layer without making direct innervation. The criteria for identifying myenteric ganglia and myenteric plexus connectives were established^8,17,41^ where at least two myenteric neurons clustered together were considered a ganglion. Two clusters of neurons were considered two ganglia if they were separated by a distance of at least three average neuron long-axis diameters, with or without the presence of a string of consecutive single-file neurons.

**Figure 1:**
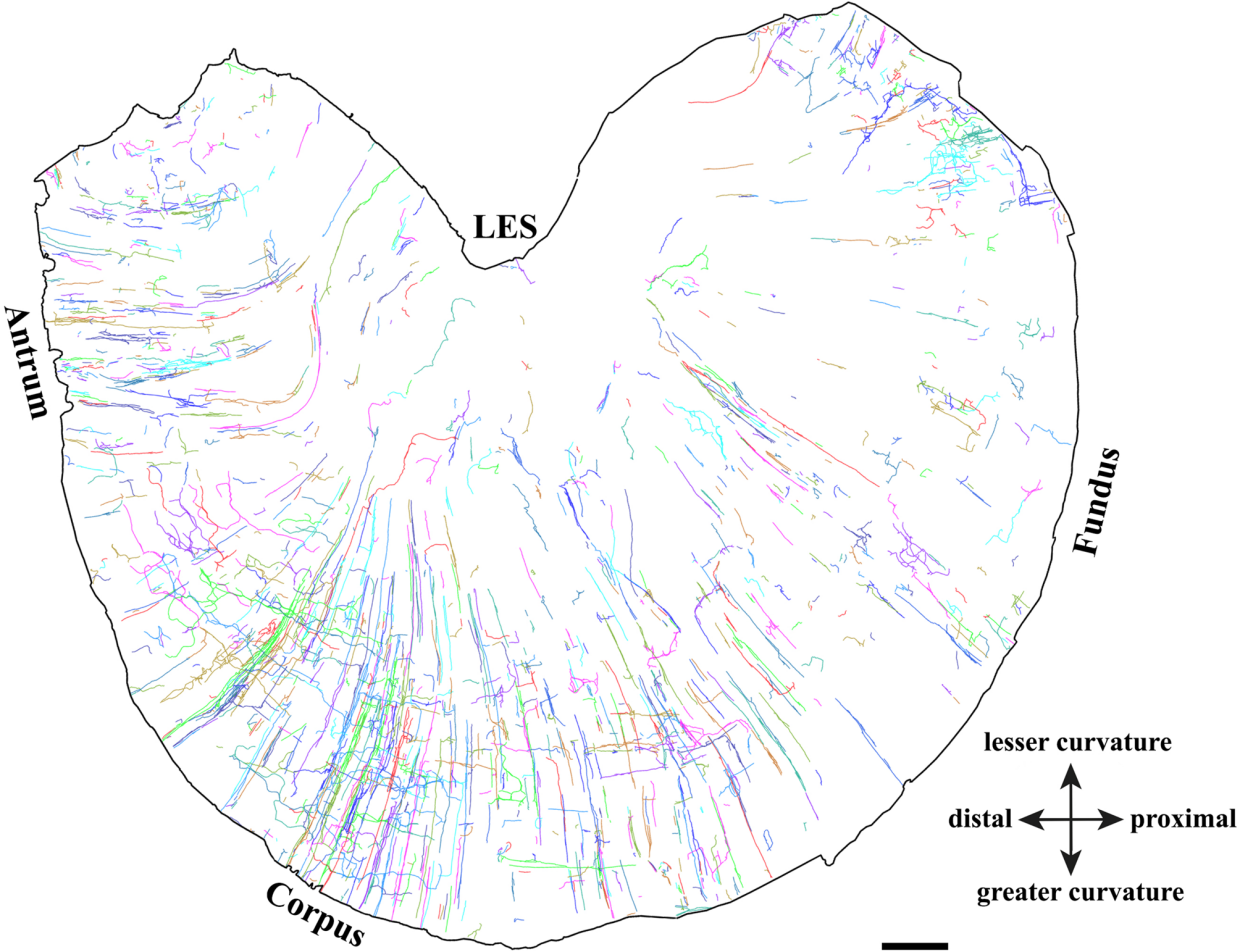
Digital reconstruction of spinal afferent axons in the muscular layers of a representative rat ventral stomach. Following the administration of tracer injections into the left DRG T7-T11, the DB-labeled axons were digitized and traced with Neurolucida 360®. The axon color was designated automatically by the software, whereby each color corresponded to a single axon that was traced. A majority of the axons ran in the myenteric plexus and along the circular/longitudinal muscle. LES = lower esophageal sphincter. Scale bar = 2 mm.

The digital reconstruction of spinal afferent axons in the stomach (i.e., Fig. 1) was performed on a 2D montage image instead of the live tissues like we mentioned above. In order to do this, we scanned the whole stomach in bright field using a 20X (NA 0.8) objective lens on a Zeiss Axio Imager M2 and performed a maximum projection for all image stacks using Zen Digital Imaging for Light Microscopy (RRID:SCR_013672, https://www.zeiss.com/microscopy/en/products/software/zeiss-zen.html). The image tiles were stitched together using Adobe Photoshop to create a montage of the whole stomach (RRID: SCR_014199, www.adobe.com/products/photoshop.html). The montage was then loaded into Neurolucida 360 (RRID:SCR_016788; MicroBrightField, Williston, VT, https://www.mbfbioscience.com/neurolucida-360) for axon tracing, digitization, and analysis. In the two-dimensional images, although branches in a reconstruction were derived from a single parent fiber, the crossing branches appear to re-unite. However, investigation in 3 dimensions showed that the crossing branches that have the appearance of re-uniting several times were in fact in different planes and did not exhibit reunification.

### Image acquisition

Single-field and multiple-field (or mosaic) photomicrographs were acquired using Zeiss Axio Imager M2 microscope with a 20X (NA 0.8) objective lens and a 63X oil immersion (NA 1.4) objective lens. The AxioCam 208 color camera was mounted on the microscope with bright field and Differential Interference Contrast (DIC) optics which introduce contrast to the images to provide definition to the smooth muscles. Mosaic photomicrographs consisted of the whole innervating field of a spinal afferent axon and were assembled via Photoshop. To capture the varying depths of a spinal afferent axon within a smooth muscle whole mount, each image consisted of multiple focal z-planes that were stacked using Photoshop to generate a partial projection image. Modifications, including brightness and contrast adjustments, and scale bar additions were conducted utilizing Photoshop or ImageJ (RRID: SCR_003070, https://imagej.net).

## Results

### General observations

Tracer-labeled spinal afferent axons from the left DRG T7-T11 were observed covering the entire stomach. Once the spinal afferent axons entered the stomach, they extensively innervated different targets (i.e., myenteric ganglia, longitudinal and circular muscular layers, blood vessels) in different regions (fundus, corpus, antrum). These axons expressed various morphological terminal structures based on the target. In the myenteric ganglia, the spinal afferent axons formed several varicose terminals around the individual neurons in the ganglia. In the muscle layers, the varicose axons ran preferentially in the muscle direction. The axons also ran along the blood vessels. Some axons may innervate both myenteric neurons within ganglia and the muscles (Fig. 1). The spinal afferent terminal structures appeared most abundantly in the muscles, followed by the myenteric ganglia and the blood vessels.

### Spinal afferent axons in the myenteric ganglia (ganglionic type)

**Figure 2:**
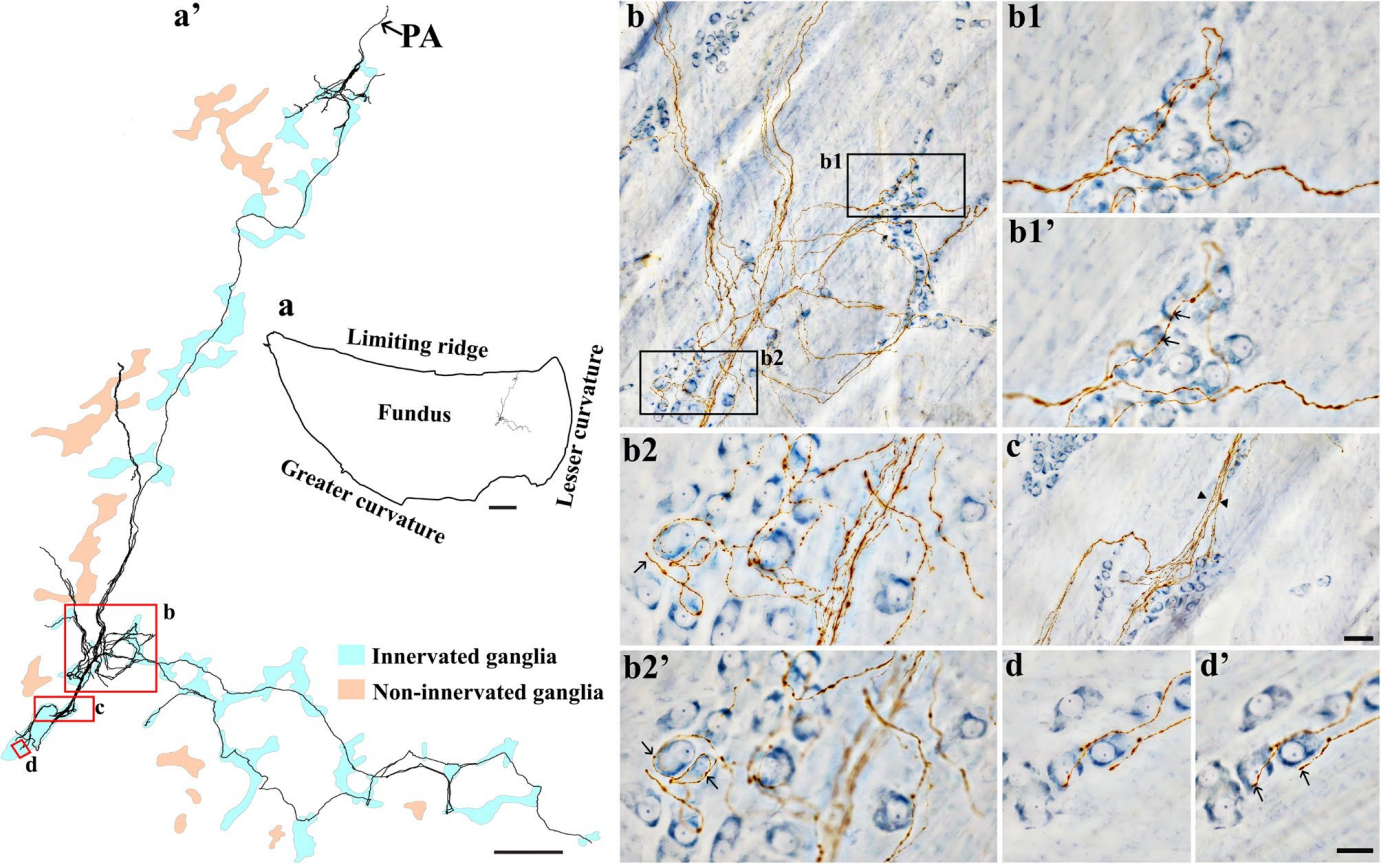
Anterograde tracer injection to the DRG specifically labeled single spinal afferent axons in myenteric ganglia of the fundus. (**a**) Contour of a representative fundus and the location of a single axon within the fundus. (**a’**) Neurolucida tracing of the axon, the innervated ganglia area (bright blue), and the non-innervated ganglia area (light orange). Innervated ganglia in this context refers to the ganglia some of whose neurons receive varicose contacts from the axon passing close to them. (**b-d**) Partial projection photomicrographs of red boxes in (**a’**) displayed the axon branching out to innervate neurons within the ganglia (**b**), traveling with the interganglionic connectives (**c**, arrowheads) and terminating in a ganglion (**d**). (**b1**, **b2**) Higher-magnification photomicrographs of black boxes in (**b**) revealed the varicose fibers either passing through (**b1**) or terminating in the ganglion (**b2**, arrow). (**b1’**, **b2’**) Single optical sections of (**b1**) and (**b2**) displayed the varicosities making contacts with the myenteric neurons (arrows). (**d’**) Single optical section of (**d**) where two terminal varicosities were observed on or beside the neurons (arrows). PA = parent axon. Scale bar in (**a**) = 3 mm, in (**a’**) = 500 μm, in (**c**) = 50 μm (also applies to **b**), in (**d’**) = 20 μm (also applies to **b1**–**b2’** and **d**).

**Figure 3:**
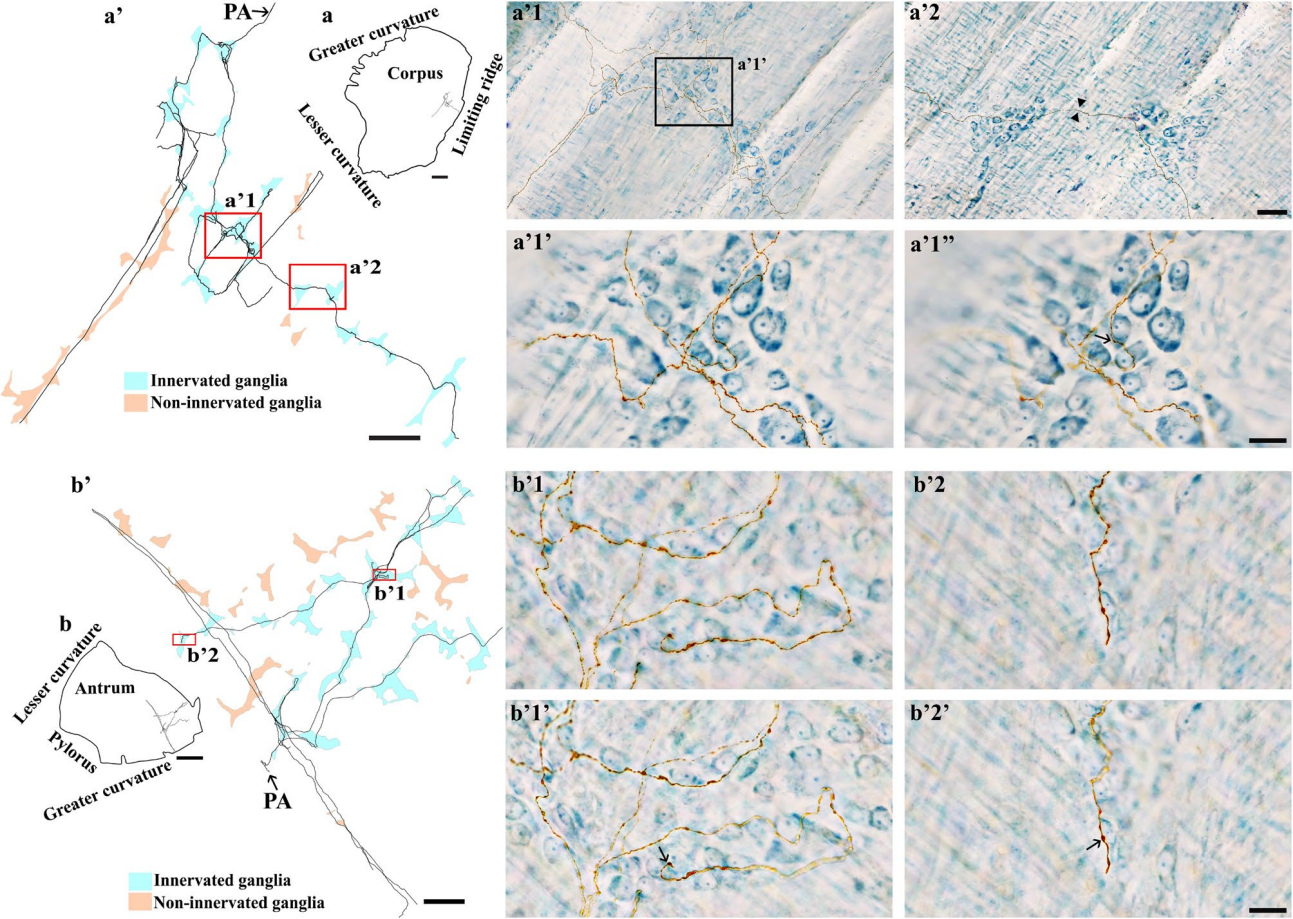
Anterograde tracer injection to the DRG specifically labeled single spinal afferent axons in myenteric ganglia of the (**a**) corpus and (**b**) antrum. (**a**, **b**) Contour of a representative corpus and antrum, respectively, and the location of a single axon within the corresponding tissue. (**a’**, **b’**) Neurolucida tracing of these axons, the innervated ganglia area (bright blue), and the non-innervated ganglia area (light orange). (**a’1**, **a’2**) Partial projection photomicrographs of red boxes in tracing (**a’**) displayed the single axon with “beads-on-a-string” varicosities traveling through the myenteric ganglion (a’1) and the interganglionic connectives (**a’2**, arrowheads). (**a’1’**, **b’1**, **b’2**) Higher-magnification photomicrographs of (**a’1**) and red boxes in tracing (b’) showed the axons traveling in close proximity to multiple neurons within the ganglia. (**a’1’’**, **b’1’**, **b’2’**) Single optical sections of (**a’1’**, **b’1**, **b’2**), respectively. The arrows point at the varicose contact of the axons on the neurons. PA = parent axon. Scale bar in (**a**, **b**) = 3 mm, in (**a’**, **b’**) = 500 μm, in (**a’2**) = 50 μm (also applies to **a’1**), in (**a’1’’**, **b’2’**) = 20 μm (also applies to **a’1’**, **b’1**, **b’1'**, **b’2**).

**Figure 4:**
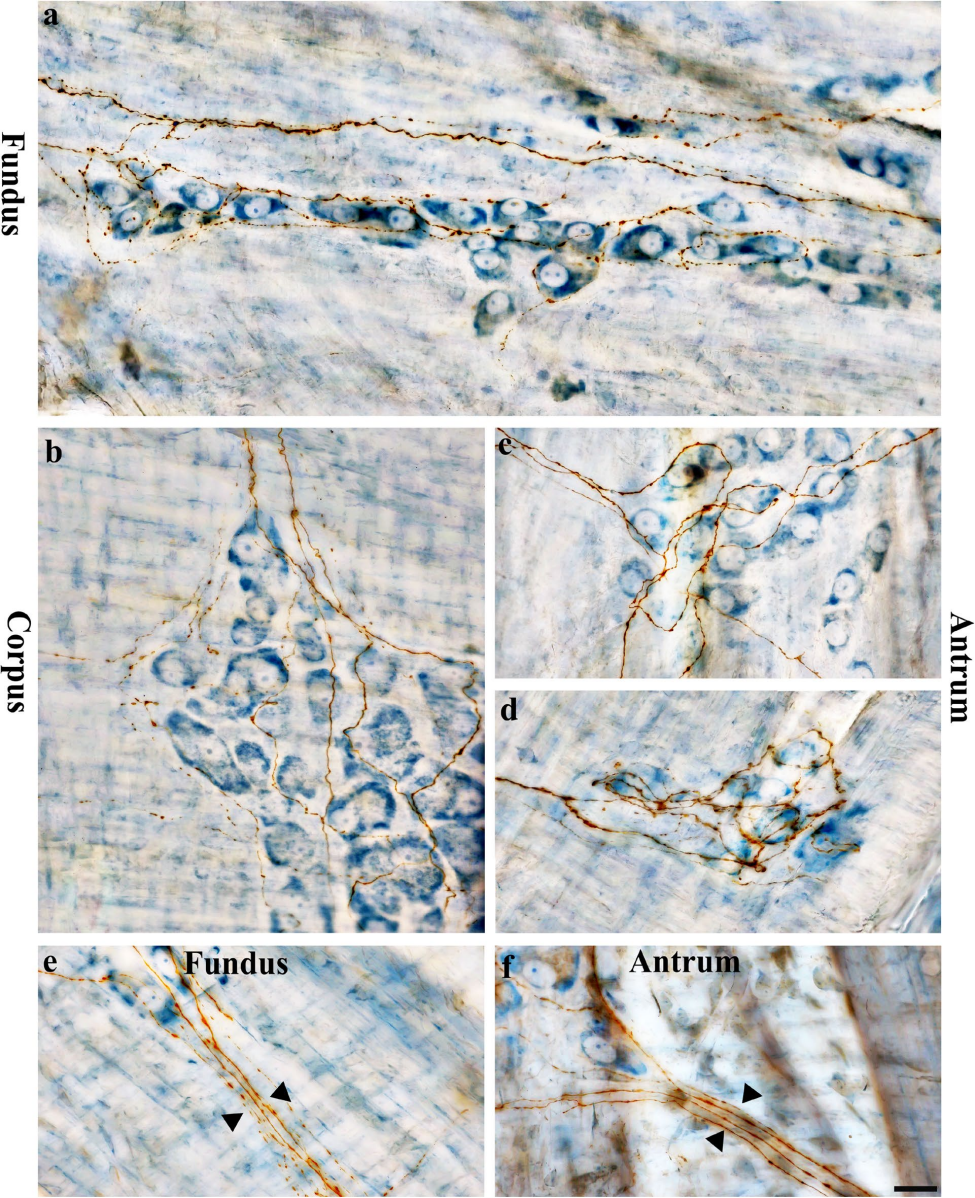
Anterogradely labeled spinal afferent axons in the myenteric plexus. (**a**–**d**) Partial projection photomicrographs depicted multiple spinal afferent axons innervating the myenteric ganglion in the fundus (**a**), corpus (**b**) and antrum (**c**, **d**). (**e**, **f**) Multiple varicose spinal axons (arrowheads) traveled with the interganglionic connectives of the fundus (**e**) and antrum (**f**). Scale bar = 20 μm.

DB injections into the DRG T7-T11 provided anterograde labeling of spinal afferent projections throughout the myenteric ganglia layer of the fundus (Fig. 2), corpus (Fig. 3a), and antrum (Fig. 3b). The network of spinal afferent projections within the ganglionic layer was apparent: the network consisted of individual axons that entered and traversed through a ganglion while supplying *en passant* varicosities to multiple myenteric neurons, as illustrated in Fig. 2b1’ (fundus) and Fig. 3a’1’’ (corpus). The axons then departed the ganglion and traveled with the interganglionic connectives (Fig. 2c, 3a’2) to innervate other ganglia. Within a ganglion, the axons weaved close to multiple neurons and subsequently formed a free terminal ending within the same ganglion, as illustrated in Fig. 2b2’ (fundus) and Fig. 3b’1’ (antrum). In some cases, the axons directly entered and terminated within the same ganglion without making any complex specializations. This structure is commonly observed and is illustrated in Fig. 2d’ (fundus) and Fig. 3b’2’ (antrum). Figure 4 displays examples of multiple spinal afferent axons that innervated the myenteric ganglia and traveled with the interganglionic connectives in different regions of the stomach.

### Spinal afferent axons in the smooth muscles (muscle type)

**Figure 5:**
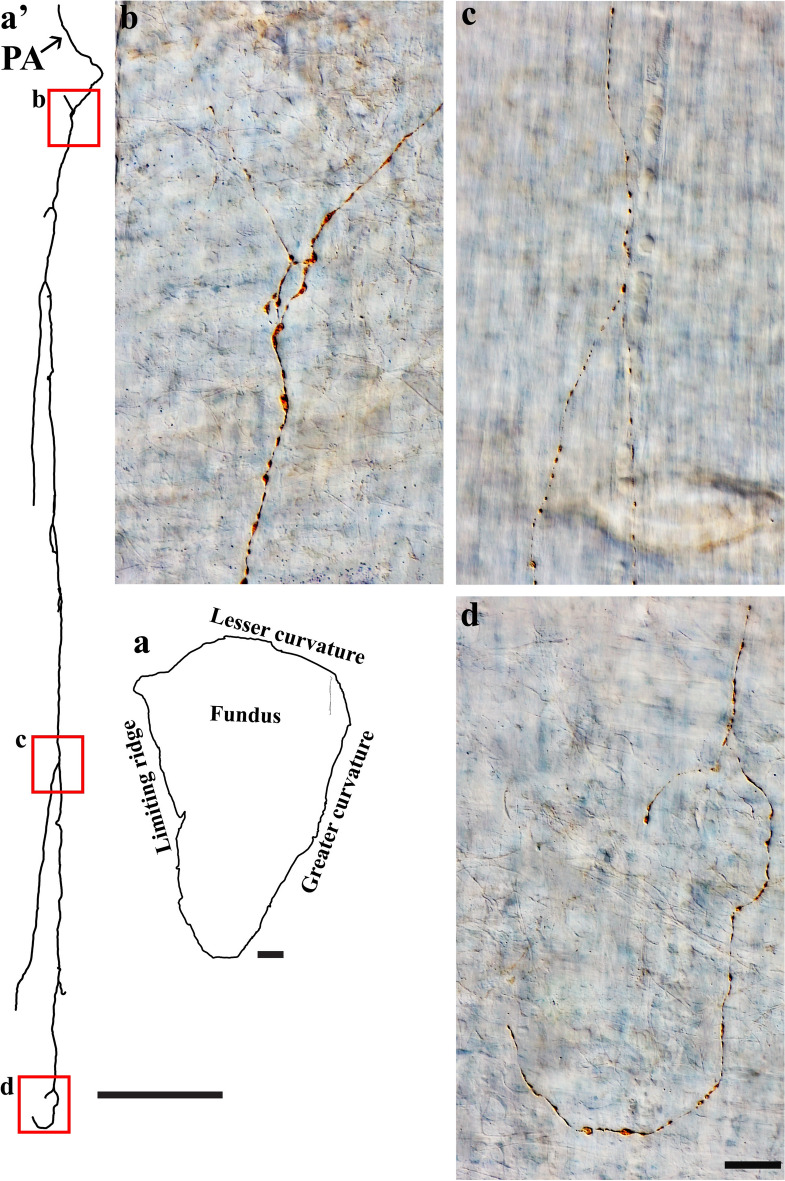
Anterogradely labeled single spinal afferent axons with several branches in the longitudinal muscle of the fundus. (**a**) Contour of a representative fundus and the location of a single axon within the fundus. (**a’**) Neurolucida tracing of this axon. (**b**–**d**) Partial projection photomicrographs of red boxes in the tracing showed the varicose axon coursing in the longitudinal muscle layer [unstained, imaged with Differential Interference Contrast (DIC)] and making several bifurcation points at different parts of its trajectory. Occasionally, a hook-like terminal ending (**d**) was observed. PA = parent axon. Scale bar in (**a**) = 3 mm, in (**a’**) = 500 μm, and in (**d**) = 20 μm (also applies to **b**, **c**).

**Figure 6:**
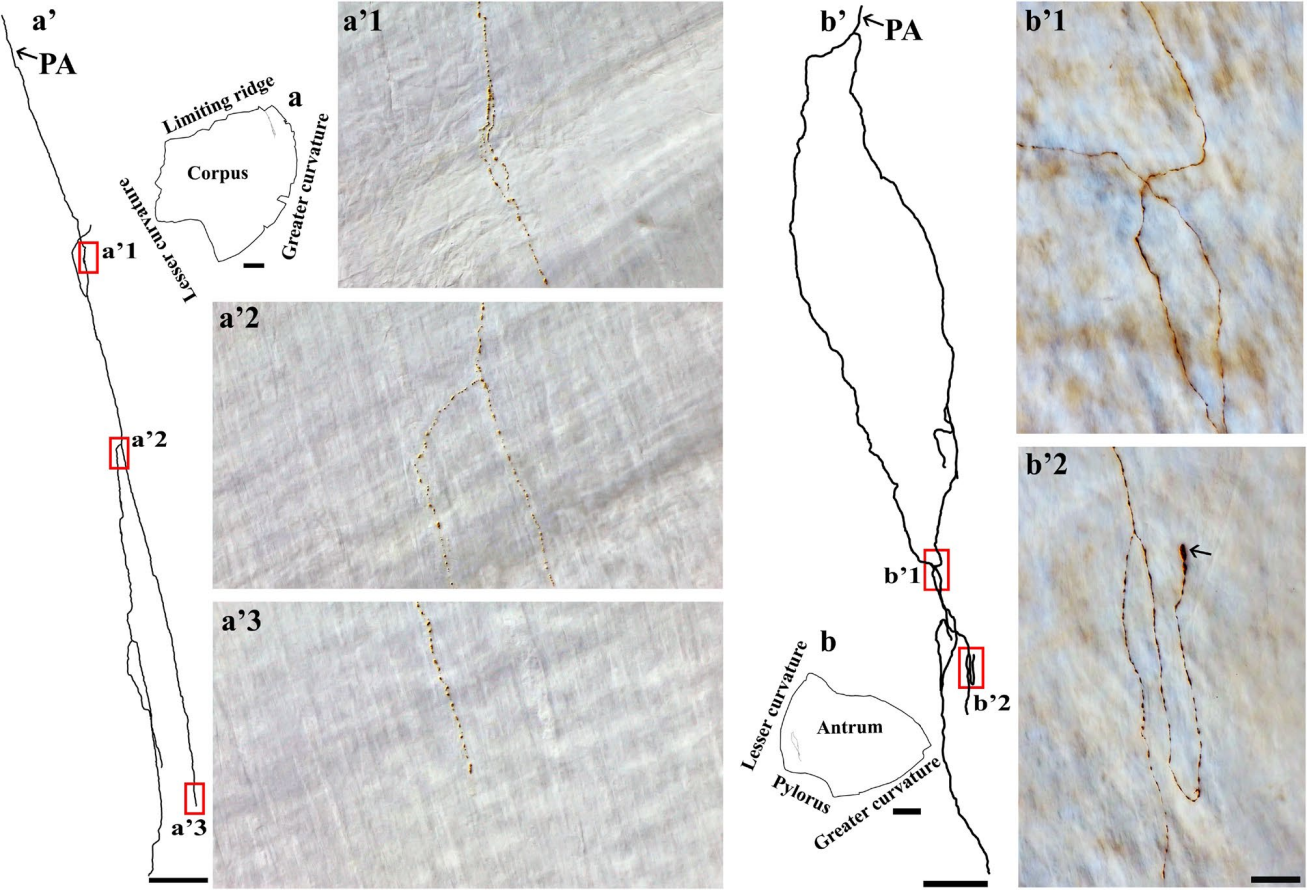
Anterogradely labeled single spinal afferent axons with several branches in the longitudinal muscle of the (**a**) corpus and (**b**) antrum. (**a**, **b**) Contour of a representative corpus and antrum and the location of a single axon within the corresponding tissue. (**a’**, **b’**) Neurolucida tracing of these axons. (**a’1**–**b’2**) Partial projection photomicrographs of red boxes in the tracings depicted the fibers running parallel to the longitudinal muscle fibers and having clear varicosities. A hooked ending with enlarged terminal varicosity was found in (**b’2**, arrow). PA = parent axon. Scale bar in (**a**, **b**) = 3 mm, in (**a’**) = 500 μm, in (**b’**) = 250 μm, in (**b’2**) = 20 μm (also applies to **a’1**–**a’3**, **b’1**).

DB-labeled individual spinal afferent axons were found in the longitudinal and circular muscles, where they coursed in the direction of the muscle fibers and expressed spherical bouton-like varicosities along the axon length. While some axons formed relatively simple bifurcations, many others ramified into several varicose branches. As shown in Fig. 5, a single spinal axon in the fundus bifurcated into several varicose branches that ran preferentially along the longitudinal muscle fibers for a long distance and formed bifurcation angles of less than 90 degrees before terminating with an enlarged varicosity. Similar observations were seen in the longitudinal muscle of the corpus (Fig. 6a) and antrum (Fig. 6b).

**Figure 7:**
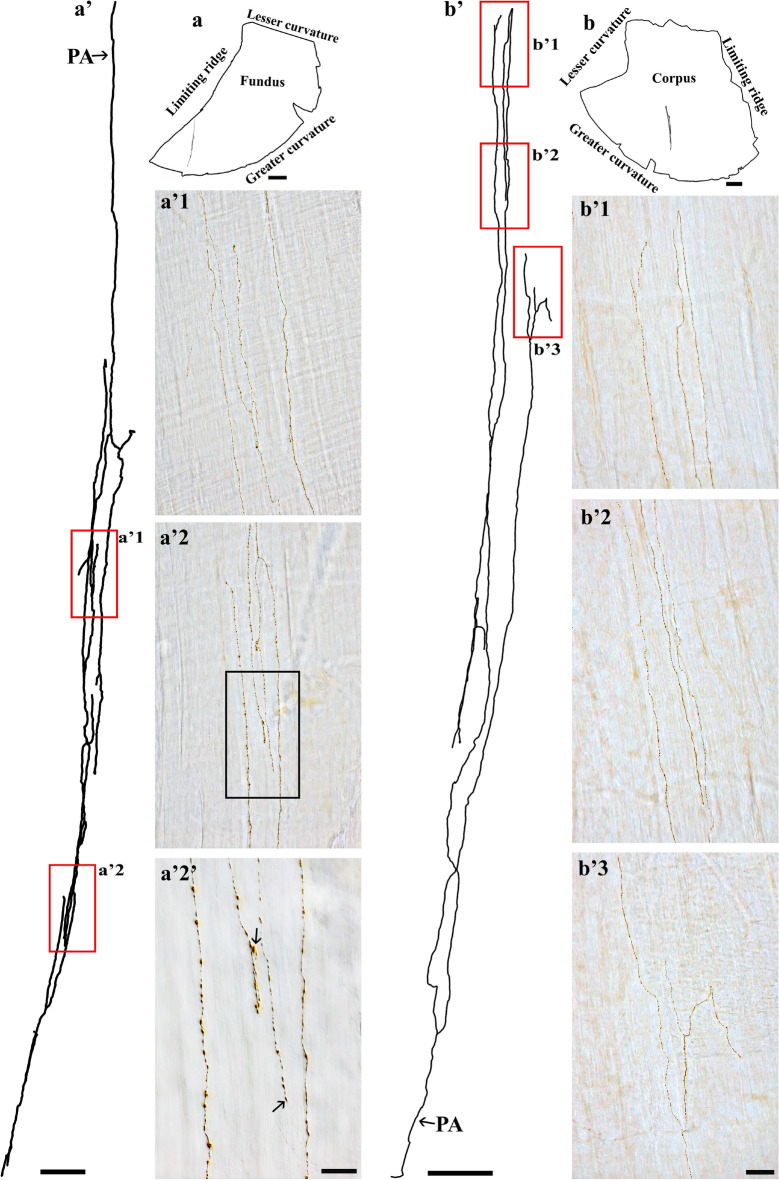
Anterogradely labeled single spinal afferent axons with several branches in the circular muscle of the (**a**) fundus and (**b**) corpus. (**a**, **b**) Contour of a representative fundus and corpus and the location of the single axon within the corresponding tissue. (**a’**, **b’**) Neurolucida tracing of these axons. (**a’1**, **a’2**, **b’1-b’3**) Partial projection photomicrographs of red boxes in (**a’**, **b’**) showed the axon frequently bifurcating into varicose branches at different points of its trajectory (**a’1**, **a’2**), making a “U” turn before termination (**b’1**), and forming a fork-like terminal ending (**b’3**). (**a’2’**) A higher-magnification photomicrograph of the black box in (**a’2**) depicted the close association of varicose branches with smooth muscles (unstained, imaged with DIC). Additionally, free terminals were observed (arrows). PA = parent axon. Scale bar in (**a**, **b**) = 3 mm, in (**a’**) = 250 μm, in (**b’**) = 500 μm, in (**a’2’**) = 20 μm, in (**b’3**) = 50 μm (also applies to **a’1**, **a’2**, **b’1**, **b’2**).

**Figure 8:**
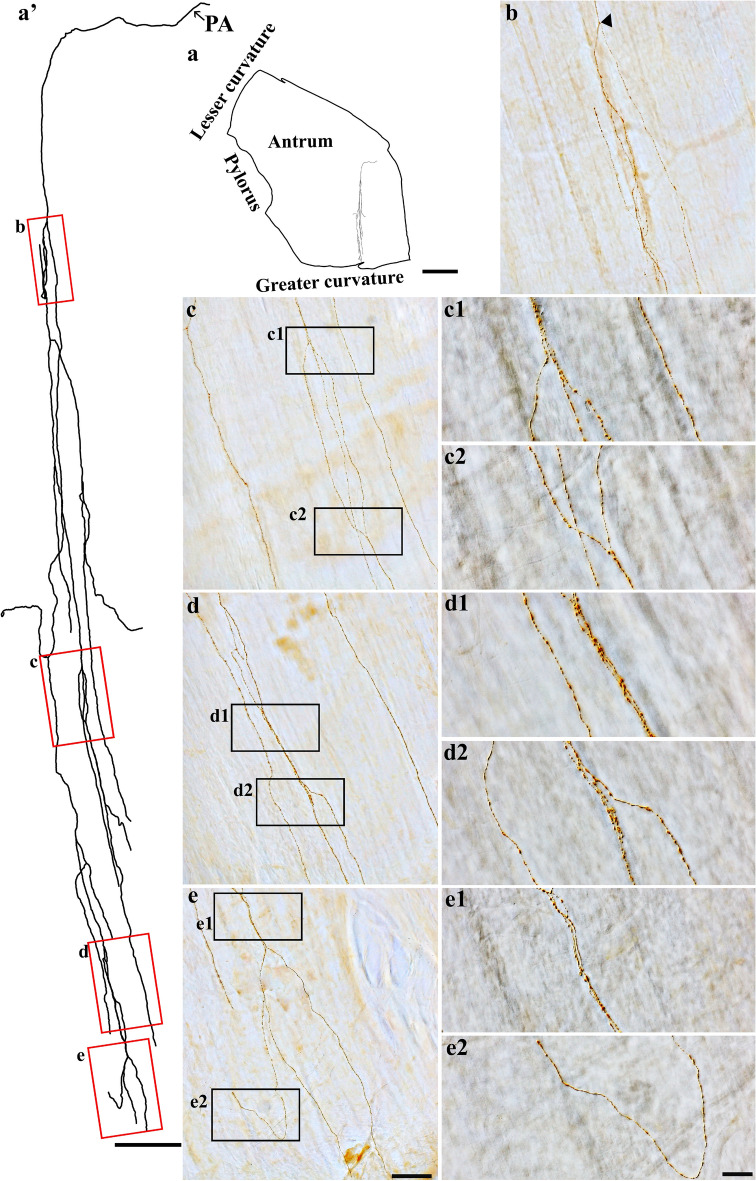
Anterogradely labeled single spinal afferent axons with several branches in the circular muscle of the antrum. (**a**) Contour of a representative antrum and the location of a single axon within the antrum. (**a’**) Neurolucida tracing of this axon. (**b**–**e**) Partial projection photomicrographs of red boxes in (**a’**) showed the axon’s first bifurcation point (**b**, arrowhead), varicose branches (**c**, **d**), and a fork-like terminal ending (**e**). (**c1**–**e2**) Higher-magnification photomicrographs of black boxes in (**c**–**e**) displayed the fibers coursing on the circular muscle layer (unstained, imaged with DIC) and expressing numerous spherical puncta-like varicosities. PA = parent axon. Scale bar in (**a**) = 3 mm, in (**a’**) = 500 μm, in (**e**) = 50 μm (also applies to **b**–**d**), and in (**e2**) = 20 μm (also applies to **c1**–**e1**).

In regard to the spinal afferent axons in the circular muscle, we found certain morphological resemblance to those found in the longitudinal muscle. Particularly, the varicose axons formed several branches in the direction of the muscle fibers and extended over a large area. This type of axon was seen more frequently in the circular muscle compared to the longitudinal muscle, perhaps as a result of the thicker circular muscle wall. For example, a single spinal axon in the fundus (Fig. 7a) and corpus (Fig. 7b) bifurcated into several varicose branches that ran parallel to each other. A similar innervation pattern was found in the antrum (Fig. 8).

**Figure 9:**
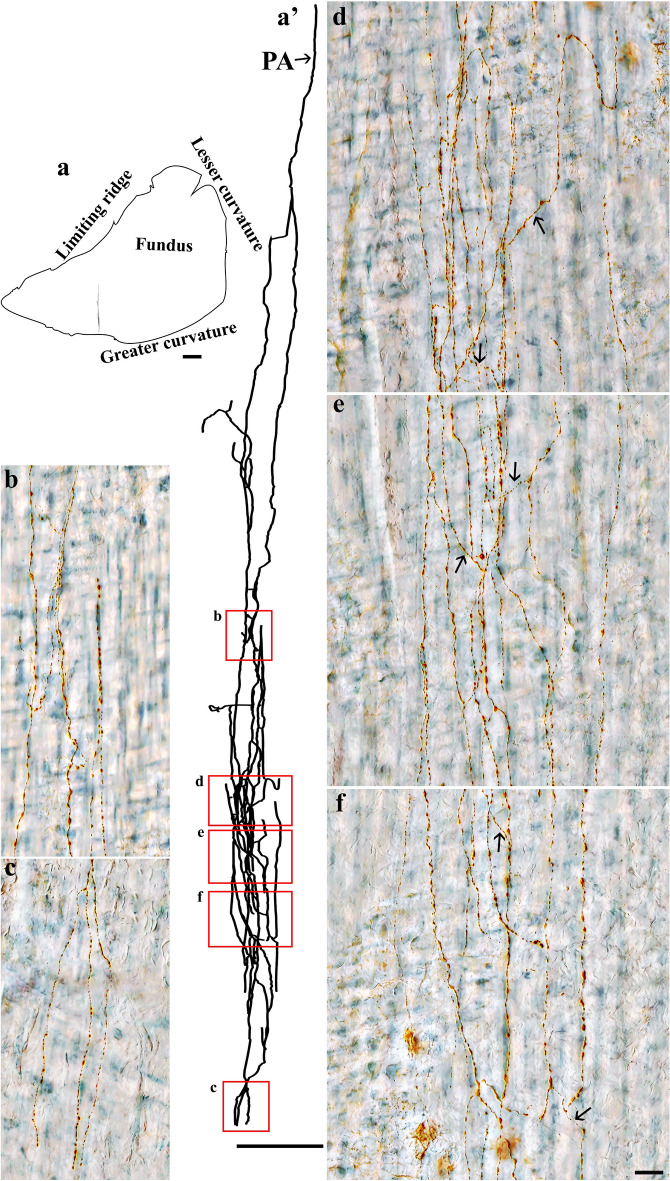
Anterogradely labeled single spinal afferent axons that arborized extensively in the fundus circular muscle. (**a**) Contour of a representative fundus and the location of a single spinal axon within the fundus. (**a’**) Neurolucida tracing of this axon. (**b**–**f**) Partial projection photomicrographs of red boxes in (**a’**) showed the branches of this arborization having numerous spherical puncta-like varicosities (**b**) and a fork-like terminal ending (**c**). This axon displayed the short crossing branches (**d**–**f**, running roughly left to right, arrows) and long main branches (running top to bottom, parallel to the muscle fibers). The tissue was stained with Cuprolinic Blue. PA = parent axon. Scale bar in (**a**) = 3 mm, in (**a’**) = 500 μm, and in (**f**) = 20 μm (also applies to **b**–**e**).

**Figure 10:**
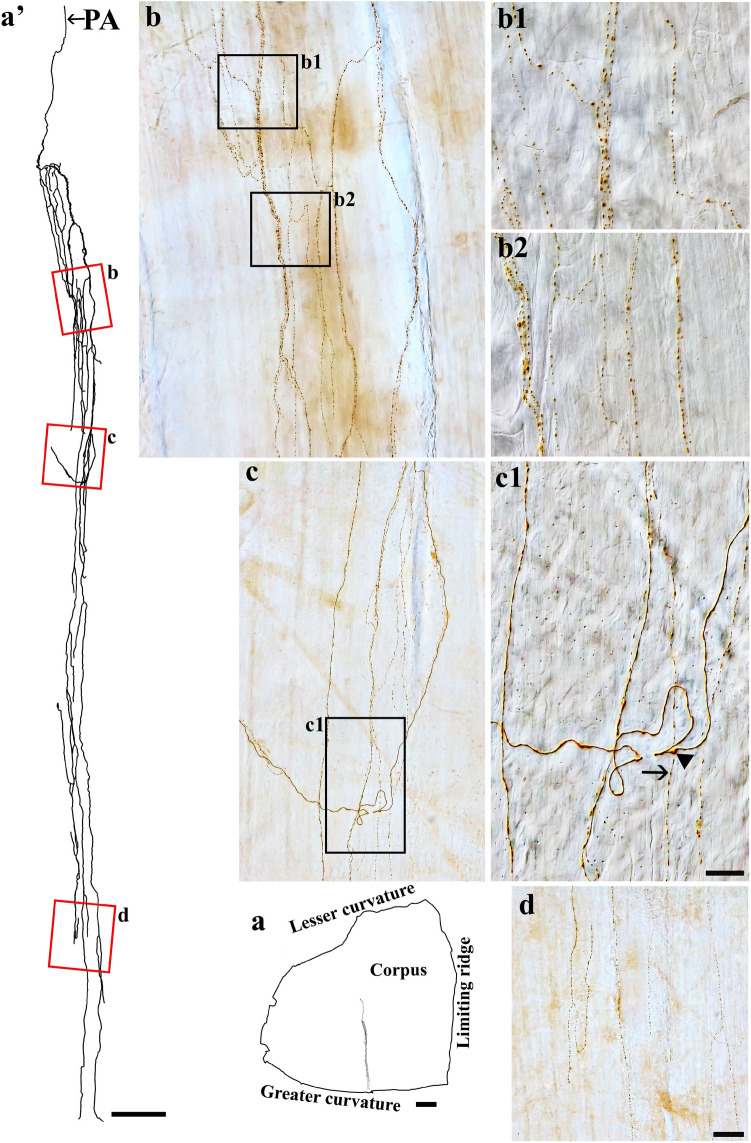
Anterogradely labeled single spinal afferent axons that arborized extensively in the corpus circular muscle. (**a**) Contour of a representative corpus and the location of a single spinal axon within the corpus. (**a’**) Neurolucida tracing of this axon. (**b**–**d**) Partial projection photomicrographs of red boxes in (**a’**). Panels (**b**, **c**) illustrated the arborizing feature of this axon and numerous spherical puncta-like varicosities distributed along the nerve fibers. (**b1**–**c1**) Higher-magnification photomicrographs of black boxes in (**b**) and (**c**) showed the close association of varicose branches with smooth muscles (unstained, imaged with DIC). Occasionally, large (arrowhead) and small (arrow) varicosities could be identified on the branches (**c1**). PA = parent axon. Scale bar in (**a**) = 3 mm, in (**a’**) = 500 μm, in (**d**) = 50 μm (also applies to **b**, **c**), and in (**c1**) = 20 μm (also applies to **b1**–**b2**).

**Figure 11:**
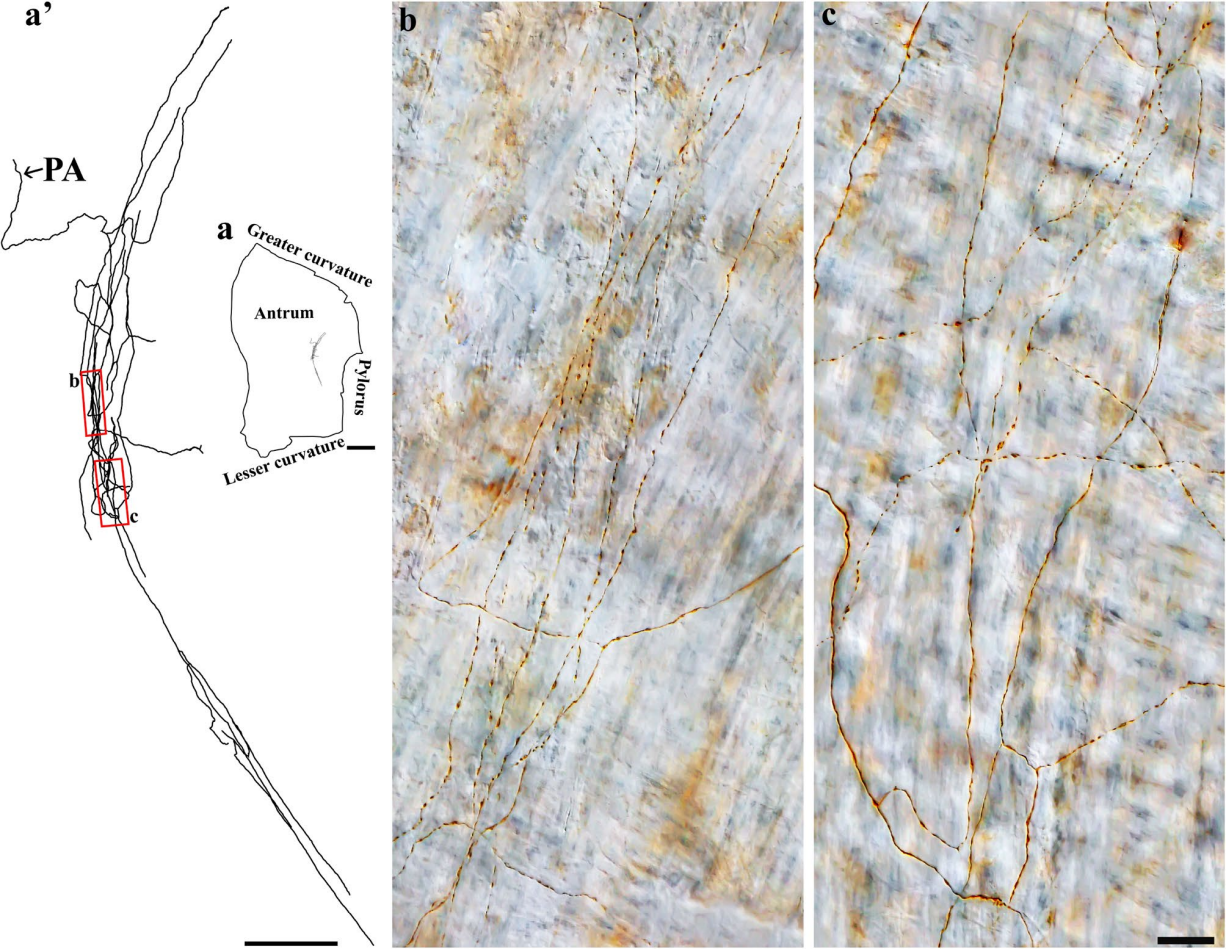
Anterogradely labeled single spinal afferent axons that arborized extensively in the antrum circular muscle. (**a**) Contour of a representative antrum and the location of a single spinal axon within the antrum. (**a’**) Neurolucida tracing of this axon. (**b**, **c**) Partial projection photomicrographs of red boxes in (**a’**). Similar to the fundus (Fig. 9) and corpus (Fig. 10), this axon contained multiple varicose branches that projected either circumferentially or longitudinally to the circular muscles (unstained, imaged with DIC). PA = parent axon. Scale bar in (**a**) = 3 mm, in (**a’**) = 500 μm, and in (**c**) = 20 μm (also applies to **b**).

In addition to the aforementioned similarities, we identified a group of individual spinal afferent axons in the circular muscle whose arborization displayed a notably higher degree of complexity. The arbor is characterized by an elongated and narrowed innervation pattern. This can be seen in Fig. 9 where a single spinal afferent axon in the fundus innervated the circular muscle by coursing in parallel with the muscle fibers. The axon then ramified extensively to form arborization whose branches ran in close proximity to each other. This spherical puncta-like varicose axon created short crossing branches and long main branches within an elongated area. A similar innervation pattern was observed in the corpus (Fig. 10) and antrum (Fig. 11).

### Spinal afferent axons in the myenteric ganglia and smooth muscles (mixed type)

**Figure 12:**
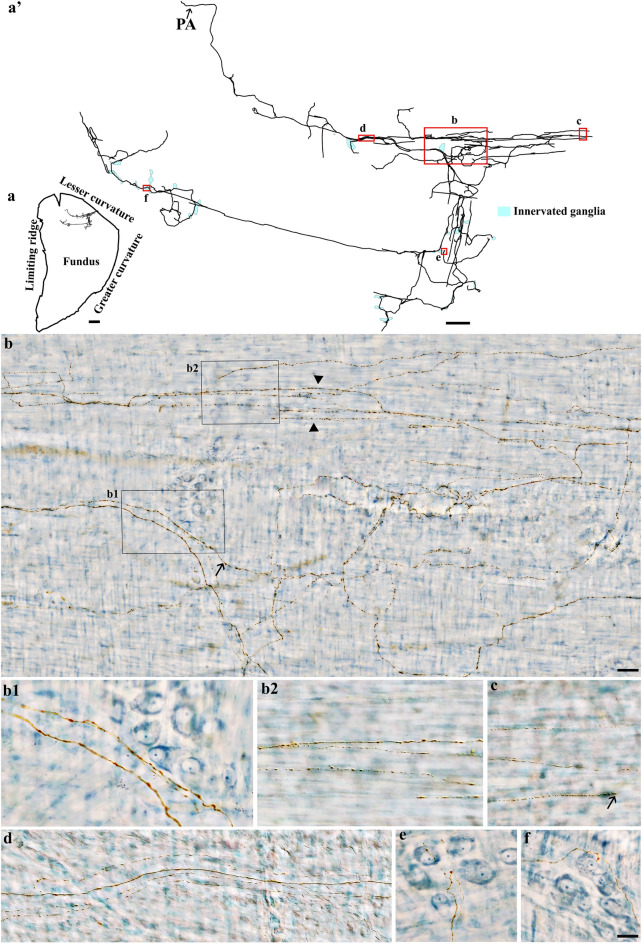
Anterogradely labeled single spinal afferent axons that co-innervated the myenteric ganglia and the circular muscle in the fundus. (**a**) Contour of a representative fundus and the location of a single spinal axon within the fundus. (**a’**) Neurolucida tracing of the axon and the innervated ganglia area (bright blue). (**b**) Partial projection photomicrograph of the red box in (**a’**) showed that the varicose fibers coursed on the circular muscle (arrowheads) with collaterals arose from the same spinal neuron innervating the myenteric plexus (arrows). (**b1**, **b2**) Single optical sections of the black boxes in (**b**) showed varicose fibers passing through the ganglion (**b1**) and coursing on the circular muscle (**b2**). (**c**, **d**) Partial projection photomicrographs of the red box in (**a’**) displayed varicose fibers terminating in the circular muscle (**c**, arrow) and traveled within the interganglionic connectives (**d**). (**e**, **f**) Single optical section images of the red box in (**a’**) showed varicose fibers innervating the myenteric ganglia. PA = parent axon. Scale bar in (**a**) = 3 mm, in (**a’**) = 500 μm, in (**b**) = 100 μm, and in (**f**) = 20 μm (also applies to **b1**, **b2**, **c**–**e**).

**Figure 13:**
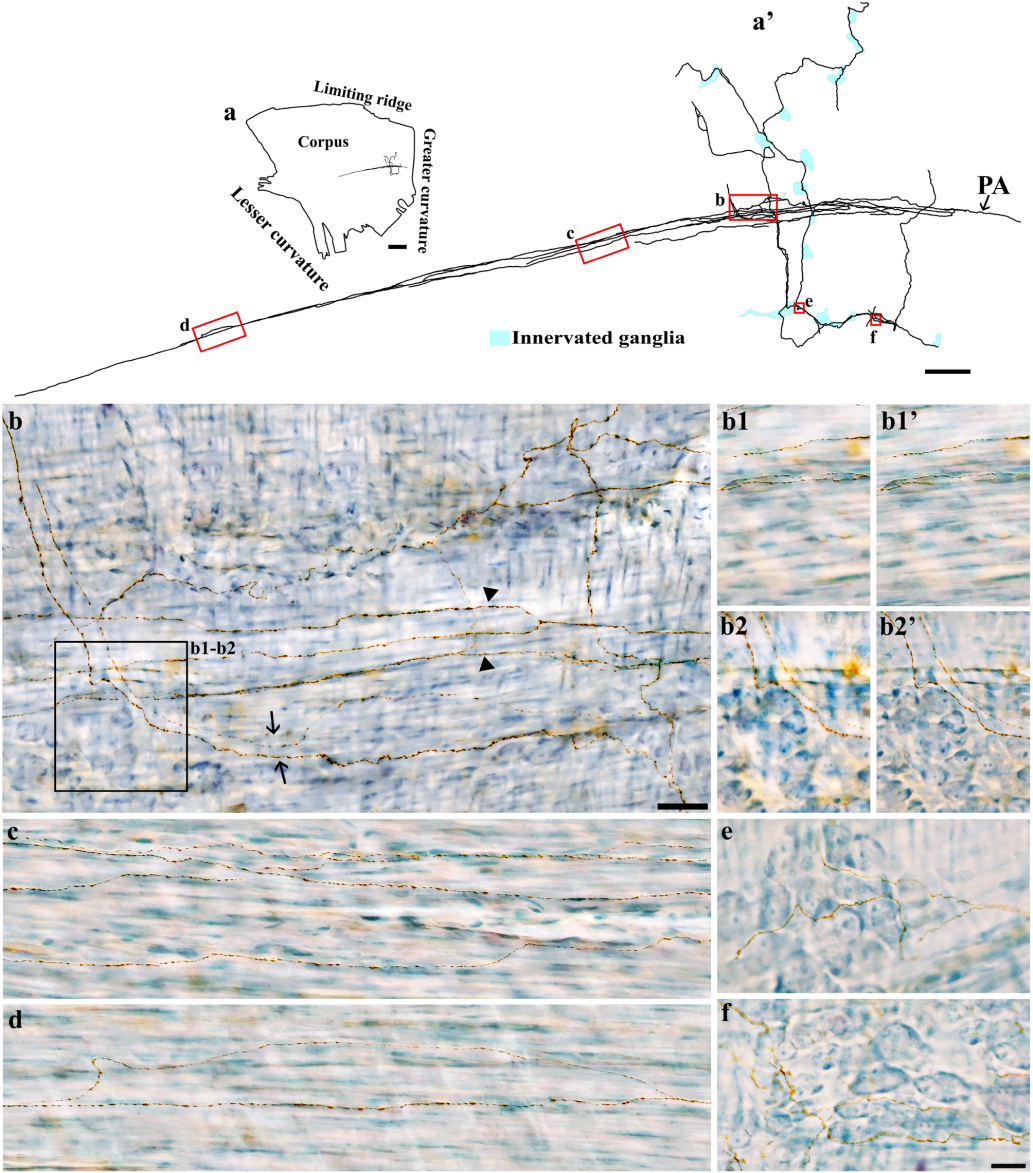
Anterogradely labeled single spinal afferent axons that co-innervated the myenteric ganglia and the circular muscle in the corpus. (**a**) Contour of a representative corpus and the location of a single spinal axon within the corpus. (**a’**) Neurolucida tracing of the axon and the innervated ganglia area (bright blue). (**b**) Partial projection photomicrograph of the red box in (**a’**) illustrated that the single axon bifurcated into many varicose branches as passageways situated in the myenteric plexus (arrows) and the circular muscle (arrowheads). (**b1**, **b2**) Partial projection photomicrographs of the black box in (**b**) showed varicose fibers innervating the circular muscle and myenteric ganglia. (**b1’**, **b2’**) Single optical sections of (**b1**) and (**b2**), respectively. (**c**–**f**) Partial projection (c, d) and single optical section (**e**, **f**) photomicrographs of red boxes in (**a’**), the axon gave off varicose branches running parallel to circular muscle fibers (**c**, **d**; unstained, imaged with DIC) and traveling through the myenteric ganglia (**e**, **f**). PA = parent axon. Scale bar in (**a**) = 3 mm, in (**a’**) = 500 μm, in (**b**) = 50 μm, and in (**f**) = 20 μm (also applies to **b1**–**b2’**, **c**–**e**).

**Figure 14:**
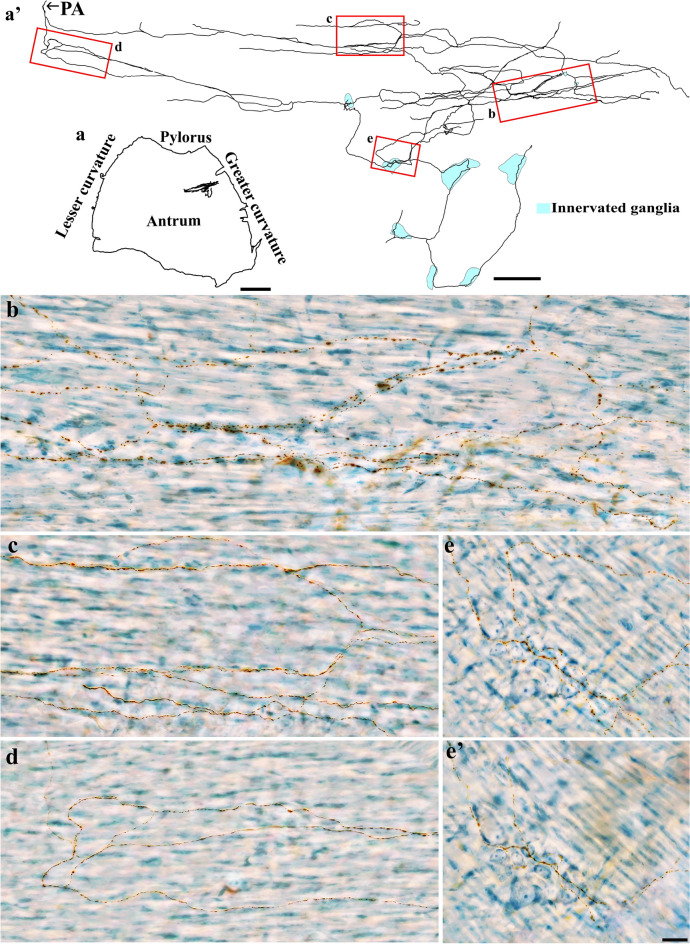
Anterogradely labeled single spinal afferent axons that co-innervated the myenteric ganglia and the circular muscle in the antrum. (**a**) Contour of a representative antrum and the location of a single spinal axon within the antrum. (**a’**) Neurolucida tracing of the axon and the innervated ganglia area (bright blue). (**b**) Partial projection photomicrograph of the red box in (**a’**) showed the varicose axon ramified through the circular muscle layer. (**c**, **d**) Partial projection photomicrographs of red boxes in (**a’**) illustrated the varicose fibers running parallel to (**c**) or in no preferential orientation to (**d**) the circular muscle fibers (unstained, imaged with DIC). (**e**) Partial projection photomicrograph of the red box in (**a’**) showed the varicose fibers innervating the myenteric ganglion. (**e’**) Single optical section of (**e**). PA = parent axon. Scale bar in (**a**) = 3 mm, in (**a’**) = 250 μm, in (**e’**) = 20 μm (apply to **b**–**e**).

Spinal afferent axons were observed to co-innervate the myenteric ganglia and circular muscle layer and also displayed different morphological terminal structures. Spinal afferent axons supplied numerous varicosities directly to multiple myenteric neurons then penetrated the circular muscle to branch repeatedly within the muscle layer. This can be seen in Fig. 12, where a single spinal afferent axon innervated the fundus by making varicose contacts with a number of myenteric ganglia, traveling within the interganglionic connectives, and ramifying extensively to innervate the circular muscle layer. During its trajectory, the varicose axon took over a large innervation field, making presumptive contacts in different tissue layers (Fig. 12b1–b2). A similar innervation pattern of spinal afferent axons was also identified in the corpus (Fig. 13) and antrum (Fig. 14). Interestingly, this type of innervation was exclusively found in the myenteric ganglia-circular muscle layers and not in myenteric ganglia-longitudinal muscle layers.

### Spinal afferent axons along the blood vessels (vascular type)

**Figure 15:**
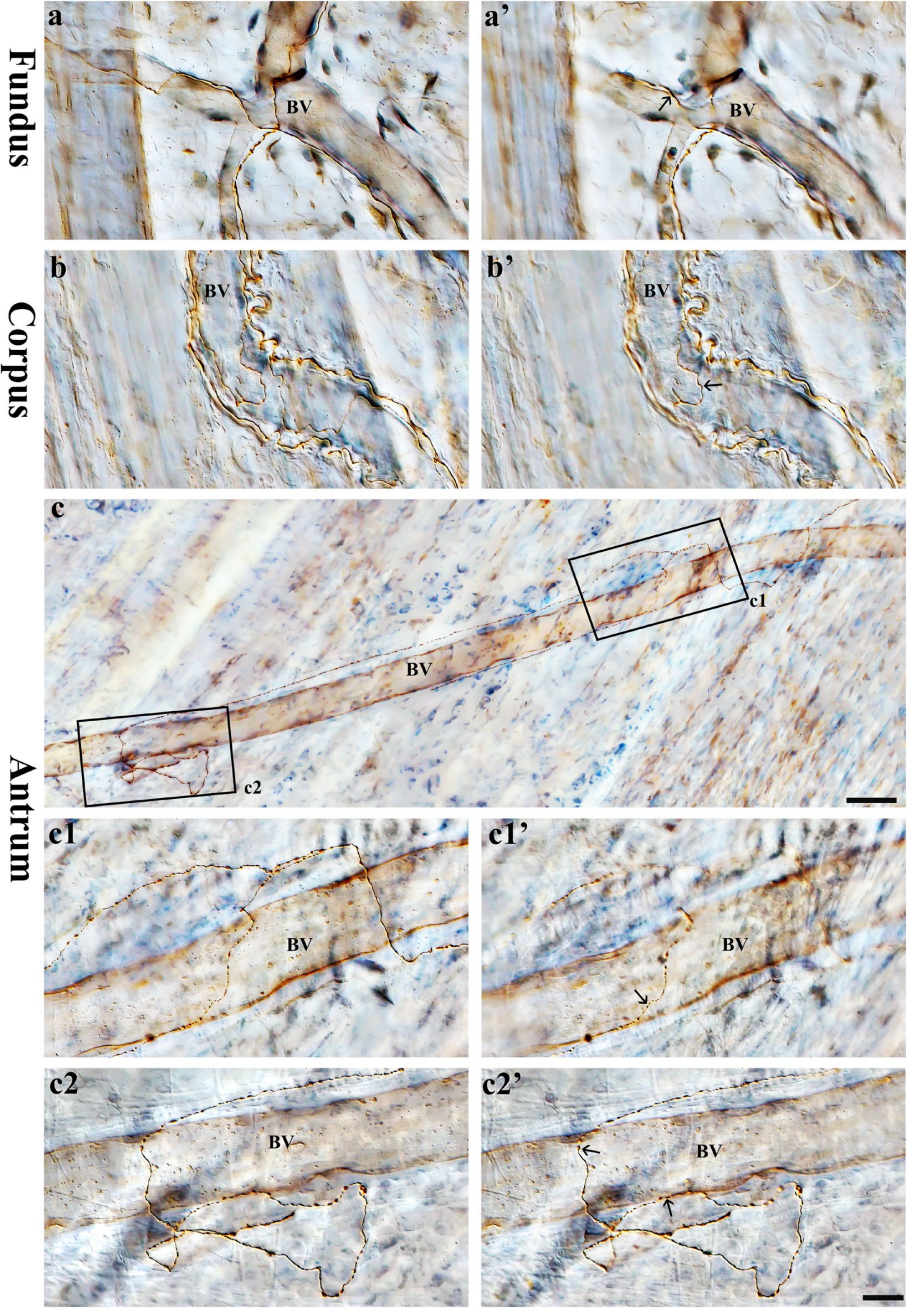
Anterograde tracer injection to the DRG specifically labeled single spinal afferent axons around the blood vessels of the fundus, corpus and antrum. (**a**–**c**) Partial projection images showed the spinal axon running across and along the blood vessel. (**c1**, **c2**) Higher-magnification photomicrographs of black boxes in (**c**). (**a’**, **b’**, **c1’**, **c2’**) Single optical sections of (**a**, **b**, **c1**, **c2**, respectively). Varicosities could be found on or close to the blood vessel walls (arrows). BV = Blood vessel. Scale bar in (**c**) = 50 μm, in (**c2’**) = 20 μm (also applies to **a-b' and c1-c2**).

The major vascular innervation is located with the ramification of the vasculature within the submucosal layers of the stomach. The separation of the submucosal layer from the muscular layer resulted in a majority of the large blood vessels being removed from the muscular layer. However, we observed spinal axons along the blood vessels in the muscle layers of the fundus, corpus, and antrum. This can be seen in Fig. 15, where DB-labeled spinal afferent axons ran parallel to and ramified over the blood vessels and had free terminals. Specifically, in the antrum (Fig. 15c–c2’), a single axon bifurcated into two branches along the blood vessel walls. These branches consisted of multiple varicosities that spanned the blood vessels and were found on or very close to the blood vessel walls. Co-innervation was not observed between muscle fibers and blood vessels or myenteric neurons and blood vessels. As a result, we believe the axons coursing along blood vessels are special vascular afferents. More observations should be made before we can come to a definitive conclusion. In the submucosal layer, we found many spinal afferent axons along the large and small blood vessels (not shown).

## Discussion

Using anterograde tracing and the Neurolucida digitization system, we characterized the spinal afferent axons and classified their terminal structures in flat-mounts of the whole stomach muscular wall. We found that tracer DB-labeled axons entered the stomach tissue and innervated myenteric ganglia, longitudinal and circular muscles and blood vessels in the fundus, corpus, and antrum. Concurrently, these spinal afferent axons formed simple, branching, and more complex terminal structures in all regions across the stomach.

### Spinal versus vagal afferent innervation of the rat stomach

Previously, Powley and his colleagues injected tracer DB (and WGA-HRP) into the nodose ganglia and characterized vagal afferent innervation in the flat mounts of the whole rat stomach muscular layers^11,12,42^. We adapted the protocol from Powley’s lab^42^ to label the spinal afferents of the whole stomach. While vagal afferents were found to innervate myenteric ganglia and muscles, spinal afferents innervated not only myenteric ganglia and muscles but also the blood vessels. We compare the similarities and differences of vagal afferent and spinal afferent innervation in the stomach as follows:

#### Spinal ganglionic type versus vagal IGLEs

Both spinal and vagal afferents innervated myenteric ganglia and formed terminal endings within those ganglia. While vagal afferents innervated the majority of myenteric ganglia across the stomach, spinal afferent axons only innervated a small proportion of myenteric ganglia. Concerning terminal morphology, vagal afferents formed extensive IGLEs that contain laminar puncta and spiny protrusions that innervated multiple neurons within a ganglion. These lamellar plates formed a large contact surface to enfold the particular neurons they innervated (Figure 3 of^24^). On the other hand, spinal afferent axons created relatively simple endings by issuing spherical varicose contacts and partially wrapping around a few individual neurons (Fig. 3). IGLEs function as tension receptors in the smooth muscle wall, and the large coverage of IGLEs on myenteric ganglia suggests their local integrated activities and information exchange with those ganglia^24^. Additionally, previous experiments using electron microscopy proposed that based on the close proximity of varicosities to neurons, these varicosities might be an indicator for synaptic contact^27,43^. Ultrastructural analysis using electron microscopy, therefore, is required to provide a more definitive assessment of the innervation of spinal afferent axons on the myenteric neurons in the future. Furthermore, the functions of spinal ganglionic type have not been studied and should be elucidated.

#### Spinal muscle type versus vagal IMAs

In the longitudinal and circular muscular layers, spinal and vagal afferent axons both preferentially ran in the direction of the smooth muscles and ramified extensively to form a variety of terminal structures. While IMAs formed complex structures in both of these muscle sheets, we only observed the spinal muscle type forming complex structures in the circular and not longitudinal muscle. Another notable difference between spinal afferents innervating the muscle and IMAs is their terminal morphology^24,42^. Vagal afferents formed IMAs that contained varicosities which varied, even in the same arbor, from relatively simple, spherical puncta to more flattened, lamellar varicosities (Figure 1 of^42^). In contrast, spinal afferents consistently formed spherical or elliptical varicosities throughout the axon length, regardless of the complexity of the branching arbors (Fig. 10). Previously, it was shown that IMAs can run in conjunction with interstitial cells of Cajal (ICC) networks^11^. The relationship between spinal afferents and ICC networks, however, has not been studied and will require further investigation.

#### Spinal versus vagal mixed type:

Both vagal and spinal afferent neurons issued single axons that innervated the myenteric ganglia and smooth muscles. The vagal mixed-type was described as IMAs in the circular muscle that sent collaterals to the myenteric ganglia. This mixed type accounts for roughly one-third of the IMAs across all three gastric regions^42^. Meanwhile, even though the spinal mixed type was also observed in all three regions, they were not as abundant as vagal afferents. Another interesting observation is that both vagal and spinal mixed type extended their innervation to myenteric ganglia and circular muscle but not myenteric ganglia and longitudinal muscle (with a single outlier of vagal mixed type innervating both myenteric ganglia and longitudinal muscle). Vagal endings in both myenteric ganglia and muscles are more complex than that of spinal endings. This form of extended arbor organization suggests a direct communication between myenteric neurons with smooth muscle sensory signaling^42^.

### Comparison of innervation patterns of spinal afferent axons in the stomach of mouse and rat

Previously, Spencer and colleagues injected dextran biotin into the DRG T8-T12 and identified different types of spinal afferents in the mouse stomach^21^. These spinal afferents range based on their endings such as intraganglionic varicose endings (IGVEs) and internodal strand endings (located in the myenteric ganglia), as well as simple-type and complex-type endings (located in the longitudinal and circular muscles). Our study utilized the rat model to demonstrate the projection of tracer-labeled spinal afferent axons in the gastric muscle wall and found, for the most part, all ending types as in the mouse stomach muscular layers. Their observations in the mouse were quite consistent with our findings in the rat: (1) Spinal afferent axons innervated the myenteric ganglia, longitudinal and circular muscles, (2) The terminal structures of spinal afferent axons in the circular muscle were more complex than that in the longitudinal muscle, (3) The terminal structures of spinal afferent axons in myenteric ganglia were somewhat simple. Alternatively, we found a substantial amount of axons in the rat stomach that displayed unique morphological structures that were not reported in the mouse. These include the single axons that innervated extensively both the circular muscle and myenteric ganglionic layers, as well as the axons that innervated the blood vessels in the muscular layers. Other studies that have specifically investigated spinal afferent innervation of blood vessels have concluded that the dense innervation is of arteries, whereas veins in the gut wall are sparsely innervated or not innervated^44^. The inclusion of blood vessels with the muscles or submucosa actually depends on different tissue peeling techniques, but the innervation of spinal afferents on the blood vessels were present in both cases. The animal size might have been a contributing factor to the increased number of axons as well as the complexity of nerve projections^45,46^.

In regard to Spencer et al.’s methodology, biotinylated dextran biotin of molecular weight 10 K was utilized to anterogradely label the spinal afferent innervation. While this strategy successfully labeled the spinal afferent network in the stomach, our experiment also made use of 3 K dextran to create a mixture of both large and small molecular weight tracers. This mixture allowed us to visualize a more comprehensive representation of innervation where not only the large axons but also the smaller and finer axons were labeled. In certain densely innervated regions, single axon tracing became quite challenging due to the overlapping of multiple axons. Using the Neurolucida system, we were able to separate and visualize the single axons and their complete trajectory in 3D, and hence more precisely track their projections. Further quantitative analysis will be needed to quantify the terminal architectures as well as the relative proportion of different types of spinal afferents in the rat stomach. Additionally, our study provided labeling of myenteric neurons using Cuprolinic Blue to distinctly characterize neurons. This counterstaining method provides both a clear profile of the myenteric neurons and evidence of the direct relationship between DB-labeled fibers and myenteric ganglia^24,40,42^.

In addition to labeling the spinal afferents with DB, Spencer et al. also performed double labeling with nociceptive marker CGRP and found that most classes of labeled nerve endings in the mouse stomach were immunoreactive to CGRP^21^. Similar to the observation in the mouse, we found many spinal afferents colocalized with CGRP (unpublished data) in the rat stomach, suggesting a possible role for spinal afferents in detecting innocuous and noxious stimuli. Overall, the resemblance between rat and mouse spinal afferent innervation suggests that the neuroanatomical features and distribution of spinal afferent axons and terminals in the stomach may be conserved, at least among closely related species. As a note, a similar conclusion was drawn by comparing vagal IGLEs and IMAs in the rat with those in the mouse^47^.

### Functional implications of spinal afferent axons in the stomach

The data of this study provides a structural map of spinal afferent innervation, and thus does not delineate the functionalities associated with said anatomical features. The functions of spinal afferents have been previously explored through electrophysiological studies, and the sensory components of the axonal processes were found to include mechanosensitive, nociceptive, chemosensitive and immunomodulatory elements^35,48,49^. Spinal afferent processes contain a diverse variety of excitatory and inhibitory neuropeptides^50,51^, which are readily released upon their corresponding stimuli through a dynamic range of intensity coding to sufficiently respond to the physiological or noxious levels^52^. As we currently do not know if different structural features might imply different functions, the use of immunohistochemical double labeling with representative nociceptive (i.e., CGRP, TRPV1)^26,53^ and mechanosensitive biomarkers (i.e., Piezo1, Piezo2)^54,55^ may offer an explanation for the physiological response of spinal afferents and more precisely elucidate the sensory, motor, and potentially protective effects of spinal afferents in healthy as well as diseased conditions such as inflammation and injury.

### Study limitations and future directions

#### Innervation in various layers of the stomach

In this study, spinal afferents within the muscular layers of the stomach were identified and classified. In addition to the types that have been identified, we believe there exists other groups of spinal afferents that innervate other layers of the stomach such as the submucosa and mucosa^21^. While the scope of this study did not go beyond the muscular layers of the stomach, it is feasible to examine the spinal afferent innervation across layers by either separating the submucosa as a whole flat-mount or utilizing sectioned tissues, both techniques were well established in our laboratory^28,56^.

#### Topographical distribution of spinal afferent innervation of the stomach

Our study described in detail the morphology and terminal structures of spinal afferent innervation of the stomach upon DB injection into the DRG. However, we have yet to conclude the laterality preference of DRG injections, meaning whether the left or right sets of DRG dominantly innervate the ventral or dorsal surfaces of the stomach, and whether they preferentially innervate certain gastric targets. Also, we have yet to evaluate the axon distribution density in different targets of different regions of the organ tissue. In order to make this study more comprehensive and topographical, a quantitative assessment and axon density analysis should be performed in the future. As mentioned above, we only selected a certain number of DRG for tracer injection, so an overlay of axon mapping data from a group of animals will, in turn, represent a normalized distribution of spinal afferents in the stomach. Future studies can also explore the similarities and/or differences of spinal afferent distribution and morphological structures in the male and female stomachs.

## Summary

In this study, state-of-the-art techniques including selective tracer injections, survival surgery procedures, bright field imaging, and axon tracing using Neurolucida were utilized to examine the detailed morphology and distribution of spinal afferent axons and their terminal structures in flat mounts of the rat stomach. Individual spinal afferent axons were found to innervate different targets of the stomach, including the myenteric ganglia, smooth muscles and blood vessels, as well as co-innervate the myenteric ganglia and smooth muscles. The results of this study provide an anatomical foundation for future studies of the functional roles that different spinal afferent terminals might have (mechanosensitive, nociceptive, chemosensitive, or immunomodulatory). In conjunction with the discoveries and implications of this research, future studies may examine the remodeling of spinal afferent innervation in pathophysiological states and the potential for therapeutic treatment of gastric diseases by selectively manipulating peripheral sensory pathways.

## Data Availability

The data used and analyzed in this study is freely available on the SPARC Portal (dataset DOI: 10.26275/rmcz-jfoq).

## References

[1] Krieger JP (2022). Neural pathway for gut feelings: Vagal interoceptive feedback from the gastrointestinal tract is a critical modulator of anxiety-like behavior. Biol. Psychiatry.

[2] Ran C (2022). A brainstem map for visceral sensations. Nature.

[3] Tsakiris M, Critchley H (2016). Interoception beyond homeostasis: Affect, cognition and mental health. Philos. Trans. R. Soc. Lond. B Biol. Sci..

[4] Cameron OG (2001). Interoception: The inside story–a model for psychosomatic processes. Psychosom. Med..

[5] Gibbins IL (1985). Co-localization of calcitonin gene-related peptide-like immunoreactivity with substance P in cutaneous, vascular and visceral sensory neurons of guinea pigs. Neurosci. Lett..

[6] Rytel L, Całka J (2016). Neuropeptide profile changes in sensory neurones after partial prepyloric resection in pigs. Ann. Anat..

[7] Rytel L, Palus K, Całka J (2015). Co-expression of PACAP with VIP, SP and CGRP in the porcine nodose ganglion sensory neurons. Anat. Histol. Embryol..

[8] Berthoud HR (1997). Distribution and structure of vagal afferent intraganglionic laminar endings (IGLEs) in the rat gastrointestinal tract. Anat. Embryol. (Berl).

[9] Berthoud HR, Powley TL (1992). Vagal afferent innervation of the rat fundic stomach: Morphological characterization of the gastric tension receptor. J. Comp. Neurol..

[10] Fox EA (2002). Selective loss of vagal intramuscular mechanoreceptors in mice mutant for steel factor, the c-Kit receptor ligand. Anat. Embryol. (Berl).

[11] Powley TL, Phillips RJ (2011). Vagal intramuscular array afferents form complexes with interstitial cells of Cajal in gastrointestinal smooth muscle: Analogues of muscle spindle organs?. Neuroscience.

[12] Wang FB, Powley TL (2000). Topographic inventories of vagal afferents in gastrointestinal muscle. J. Comp. Neurol..

[13] Zagorodnyuk VP, Chen BN, Brookes SJ (2001). Intraganglionic laminar endings are mechano-transduction sites of vagal tension receptors in the guinea-pig stomach. J. Physiol..

[14] Berthoud HR, Neuhuber WL (2000). Functional and chemical anatomy of the afferent vagal system. Auton. Neurosci..

[15] Brookes SJ (2013). Extrinsic primary afferent signalling in the gut. Nat. Rev. Gastroenterol. Hepatol..

[16] Tan LL, Bornstein JC, Anderson CR (2009). Neurochemical and morphological phenotypes of vagal afferent neurons innervating the adult mouse jejunum. Neurogastroenterol. Motil..

[17] Walter GC (2016). Individual sympathetic postganglionic neurons coinnervate myenteric ganglia and smooth muscle layers in the gastrointestinal tract of the rat. J. Comp. Neurol..

[18] Walter GC (2009). Versatile, high-resolution anterograde labeling of vagal efferent projections with dextran amines. J. Neurosci. Methods.

[19] Powley TL (2013). Vagal afferent innervation of the lower esophageal sphincter. Auton. Neurosci..

[20] Powley TL (2014). Organization of vagal afferents in pylorus: Mechanoreceptors arrayed for high sensitivity and fine spatial resolution?. Auton. Neurosci..

[21] Spencer NJ (2016). Different types of spinal afferent nerve endings in stomach and esophagus identified by anterograde tracing from dorsal root ganglia. J. Comp. Neurol..

[22] Costa M, Brookes SH, Zagorodnyuk V (2004). How many kinds of visceral afferents?. Gut.

[23] Furness JB (2014). The enteric nervous system and gastrointestinal innervation: Integrated local and central control. Adv. Exp. Med. Biol..

[24] Powley TL (2019). Vagal innervation of the stomach reassessed: Brain-gut connectome uses smart terminals. Ann. N. Y. Acad. Sci..

[25] Uesaka T (2016). Development of the intrinsic and extrinsic innervation of the gut. Dev. Biol..

[26] Spencer NJ (2016). Spinal afferent nerve endings in visceral organs: Recent advances. Am. J. Physiol. Gastrointest. Liver Physiol..

[27] Mazzia C, Clerc N (1997). Ultrastructural relationships of spinal primary afferent fibres with neuronal and non-neuronal cells in the myenteric plexus of the cat oesophago-gastric junction. Neuroscience.

[28] Ma J (2022). Topographical organization and morphology of substance P (SP)-immunoreactive axons in the whole stomach of mice. J. Comp. Neurol..

[29] Tarif AMM (2021). Immunohistochemical expression and neurochemical phenotypes of huntingtin-associated protein 1 in the myenteric plexus of mouse gastrointestinal tract. Cell Tissue Res..

[30] Ma J, Nguyen D, Madas J, Bizanti A, Mistareehi A, Kwiat AM, Chen J, Lin M, Christie R, Hunter P, Heal M, Baldwin S, Tappan S, Furness JB, Powley TL, Cheng ZJ (2023). Organization and morphology of calcitonin gene-related peptide-immunoreactive axons in the whole mouse stomach. J Comp Neurol..

[31] Palus K, Bulc M, Calka J (2018). Changes in VIP-, SP- and CGRP- like immunoreactivity in intramural neurons within the pig stomach following supplementation with low and high doses of acrylamide. Neurotoxicology.

[32] Zalecki M (2019). Gastric ulcer induced changes in substance P and Nk1, Nk2, Nk3 receptors expression in different stomach localizations with regard to intrinsic neuronal system. Histochem. Cell Biol..

[33] Clerc N, Mazzia C (1994). Morphological relationships of choleragenoid horseradish peroxidase-labeled spinal primary afferents with myenteric ganglia and mucosal associated lymphoid tissue in the cat esophagogastric junction. J. Comp. Neurol..

[34] Mazzia C, Clerc N (2000). Ultrastructural analysis of spinal primary afferent fibers within the circular muscle of the cat lower esophageal sphincter. Histochem. Cell Biol..

[35] Dodds KN (2022). The gut-brain axis: Spatial relationship between spinal afferent nerves and 5-HT-containing enterochromaffin cells in mucosa of mouse colon. Am. J. Physiol. Gastrointest Liver Physiol.

[36] Jaffey DM (2023). Vagal preganglionic axons arborize in the myenteric plexus into two types: nitrergic and non-nitrergic postganglionic motor pools?. Am. J. Physiol. Regul. Integr. Comp. Physiol..

[37] Gómez-Álvarez M, Saldaña E (2016). Different tonotopic regions of the lateral superior olive receive a similar combination of afferent inputs. J. Comp. Neurol..

[38] Reiner A (2000). Pathway tracing using biotinylated dextran amines. J. Neurosci. Methods.

[39] Takiguchi M (2015). Compensatory projections of primary sensory fibers in lumbar spinal cord after neonatal thoracic spinal transection in rats. Neuroscience.

[40] Phillips RJ (2004). Quantification of neurons in the myenteric plexus: An evaluation of putative pan-neuronal markers. J. Neurosci. Methods.

[41] Bar-Shai A (2004). Decreased density of ganglia and neurons in the myenteric plexus of familial dysautonomia patients. J. Neurol. Sci..

[42] Powley TL (2016). Vagal intramuscular arrays: The specialized mechanoreceptor arbors that innervate the smooth muscle layers of the stomach examined in the rat. J. Comp. Neurol..

[43] Powley TL (2008). Ultrastructural evidence for communication between intramuscular vagal mechanoreceptors and interstitial cells of Cajal in the rat fundus. Neurogastroenterol. Motil..

[44] Furness JB (1982). Substance P-like immunoreactivity in nerves associated with the vascular system of guinea-pigs. Neuroscience.

[45] Purves D, Lichtman JW (1985). Geometrical differences among homologous neurons in mammals. Science.

[46] Purves D (1986). Relation of animal size to convergence, divergence, and neuronal number in peripheral sympathetic pathways. J. Neurosci..

[47] Fox EA (2000). Vagal afferent innervation of smooth muscle in the stomach and duodenum of the mouse: morphology and topography. J. Comp. Neurol..

[48] Brierley SM, Hibberd TJ, Spencer NJ (2018). Spinal afferent innervation of the colon and rectum. Front. Cell Neurosci..

[49] Kyloh M (2011). Identification of the visceral pain pathway activated by noxious colorectal distension in mice. Front. Neurosci..

[50] de Groat WC (1987). Neuropeptides in pelvic afferent pathways. Experientia.

[51] Kawatani M, Nagel J, de Groat WC (1986). Identification of neuropeptides in pelvic and pudendal nerve afferent pathways to the sacral spinal cord of the cat. J. Comp. Neurol..

[52] Grundy D (2006). Signalling the state of the digestive tract. Auton. Neurosci..

[53] Numazaki M, Tominaga M (2004). Nociception and TRP Channels. Curr. Drug Targets CNS Neurol. Disord..

[54] Coste B (2010). Piezo1 and Piezo2 are essential components of distinct mechanically activated cation channels. Science.

[55] Romac JM (2018). Piezo1 is a mechanically activated ion channel and mediates pressure induced pancreatitis. Nat. Commun..

[56] Mistareehi A (2023). Topographical distribution and morphology of SP-IR axons in the antrum, pylorus, and duodenum of mice. Auton. Neurosci..

